# Identification of the transcription factor Miz1 as an essential regulator of diphthamide biosynthesis using a CRISPR-mediated genome-wide screen

**DOI:** 10.1371/journal.pgen.1009068

**Published:** 2020-10-15

**Authors:** Jie Liu, Zehua Zuo, Meijuan Zou, Toren Finkel, Shihui Liu

**Affiliations:** 1 Aging Institute of University of Pittsburgh and University of Pittsburgh Medical Center, Pittsburgh, PA, United States of America; 2 Division of Cardiology, Department of Medicine, University of Pittsburgh School of Medicine, Pittsburgh, PA, United States of America; 3 Division of Infectious Diseases, Department of Medicine, University of Pittsburgh School of Medicine, Pittsburgh, PA, United States of America; Walter and Eliza Hall Institute of Medical Research, AUSTRALIA

## Abstract

Diphthamide is a unique post-translationally modified histidine residue (His^715^ in all mammals) found only in eukaryotic elongation factor-2 (eEF-2). The biosynthesis of diphthamide represents one of the most complex modifications, executed by protein factors conserved from yeast to humans. Diphthamide is not only essential for normal physiology (such as ensuring fidelity of mRNA translation), but is also exploited by bacterial ADP-ribosylating toxins (e.g., diphtheria toxin) as their molecular target in pathogenesis. Taking advantage of the observation that cells defective in diphthamide biosynthesis are resistant to ADP-ribosylating toxins, in the past four decades, seven essential genes (*Dph1* to *Dph7)* have been identified for diphthamide biosynthesis. These technically unsaturated screens raise the question as to whether additional genes are required for diphthamide biosynthesis. In this study, we performed two independent, saturating, genome-wide CRISPR knockout screens in human cells. These screens identified all previously known *Dph* genes, as well as further identifying the BTB/POZ domain-containing transcription factor Miz1. We found that Miz1 is absolutely required for diphthamide biosynthesis via its role in the transcriptional regulation of *Dph1* expression. Mechanistically, Miz1 binds to the *Dph1* proximal promoter via an evolutionarily conserved consensus binding site to activate Dph1 transcription. Therefore, this work demonstrates that Dph1-7, along with the newly identified Miz1 transcription factor, are likely to represent the essential protein factors required for diphthamide modification on eEF2.

## Author summary

Diphthamide is a unique post-translationally modified histidine residue (His^699^ in yeast, His^715^ in all mammals) found only in eukaryotic elongation factor-2 (eEF-2). Mice that are deficient in diphthamide biosynthesis are embryonic lethal, attesting to the importance of diphthamide in normal physiology. It has taken four decades to identify the seven non-redundant genes in diphthamide biosynthesis, but whether additional factors are required and how the pathway is regulated remained elusive. To address these issues, we performed two saturating, independent, and unbiased genome-wide CRISPR knockout screens. The screens concluded independently that Dph1-Dph7 and additionally transcription factor Miz1 are the key factors required for diphthamide biosynthesis. Mechanistically, Miz1 binds to the Dph1 proximal promoter via an evolutionarily conserved consensus binding site to activate Dph1 transcription. While diphthamide biosynthesis machinery (Dph1-Dph7) exists across eukaryotes, Miz1 orthologues do not exist in lower species such as yeast, C. elegans, and Drosophila, indicating that the regulation of diphthamide modification by Miz1 emerged much later in evolution. The work opens a new avenue for understanding the role that diphthamide modification plays in normal physiology and bacterial toxin pathogenesis.

## Introduction

Bacterial pathogens have evolved to use various exotoxins to target a variety of pathways of mammalian host target cells for their pathogenesis. Investigation on the interaction of these toxins with their target cells has not only demonstrated the mechanisms underlying toxins’ action but also often provided new insights in understanding of those pathways modified by toxins. One example was the discovery of the diphthamide modification on eukaryotic translation elongation factor-2 (eEF2), resulting from studying the pathogenic action of *Corynebacterium dipheriae* diphtheria toxin (DT) [1–3].

Diphthamide is a uniquely post-translationally modified histidine residue (His^699^ in yeast, His^715^ in all mammals) found only in eEF-2, that is exploited as the molecular target by DT and other bacterial ADP-ribosylating (ADPR) toxins including *Pseudomonas aeruginosa* exotoxin A (ETA) and *Vibrio cholerae* cholix toxin [1, 4]. These ADPR toxins specifically catalyze the transfer of ADP-ribose from NAD^+^ (nicotinamide adenine dinucleotide) to diphthamide (S1 Fig), thus inactivating eEF-2, halting cellular protein synthesis, and causing cell death. Although diphthamide has been found in all eukaryotic organisms and archaea, it is not present in eubacteria. Thus, DT and ETA have evolved a specific mechanism for targeting the eukaryotic protein synthesizing machinery without inactivating the analogous elongation factor (EF-G) present in the bacterial pathogens that produce them.

The biosynthesis of diphthamide, which is conserved from yeast to humans, represents one of the most complex known post-translational modifications. The biosynthesis is accomplished by stepwise additions and modifications of a three-carbon-side chain to the His^715^ (His^699^ in yeast) residue of eEF-2 [1, 5–8] (S1 Fig). Mutants defective in diphthamide biosynthesis have been isolated in both CHO cells and yeast cells by selection for resistance to the action of DT and ETA. While these cells exhibit no obvious growth phenotypes [5, 6], mice lacking the capacity for diphthamide modification are embryonic lethal, attesting to the importance of this pathway in normal physiology [9–12]. Recently, we and others have shown that diphthamide modification plays a critical role in maintaining eEF2 in an appropriate conformation to assure fidelity of mRNA translation, as well as for maintaining full activity for protein synthesis [12, 13]. Therefore, diphthamide is crucial in maintaining cell fitness and responsiveness to a variety of cellular stresses, including oxidative stress [14–17].

In the past four decades since the discovery of diphthamide modification, tremendous efforts have been made to reveal the key genes required for diphthamide biosynthesis. These efforts have cumulatively identified seven genes, namely *Dph1* to *Dph7*, required for diphthamide modification on eEF2 [5, 6, 18–24]. However, due to the limitations of each genetic approach used in these studies, including retroviral insertional mutagenesis, gene-trapping, and data mining, only a subset of these genes were identified in each study. For example, only haploid genes could be identified using the insertional mutagenesis and gene-trapping. Therefore, these screens, which were unsaturated in nature, raise the question as to whether additional genes may be required for diphthamide biosynthesis. Further, how diphthamide biosynthesis is regulated under various physiological conditions remains unknown. In this study, we exploited the utility of a genome-wide CRISPR knockout (KO) screen to attempt to define the essential factors required for regulation and biosynthesis of diphthamide. As such, this work provides additional insights into the mechanisms underlying diphthamide biosynthesis and the role this modification plays in normal physiology.

## Results

### Genome-wide CRISPR KO screen

To circumvent the limitations of the genetic approaches used previously, we performed unbiased and saturating genome-wide CRISPR knockout screens in human HT1080 cells to address whether additional factors/regulators are required for diphthamide biosynthesis. We reasoned that following CRISPR-based gene editing, cells with functionally inactivated genes essential for diphthamide biosynthesis would exhibit resistance to ADPR toxins. This would be reflected in an overall survival advantage after toxin treatment. We used the potent, modified anthrax lethal toxin, consisting of PA (anthrax protective antigen) and FP59 (ETA fusion protein 59), as the toxin for genetic selection. The native anthrax lethal toxin comprises the cell binding and delivery component–PA and the enzymatic moiety–LF (lethal factor). LF is a Zn^2+^-dependent metalloproteinase that cleaves and inactivates the mitogen-activated protein kinase kinases (MEKs) [25–28]. FP59 is a fusion protein of LFn (N-terminal PA binding domain of LF) and the catalytic domain of ETA that kills all cells by ADP-ribosylation of eEF2’s diphthamide following PA-mediated delivery into the cytosol [29]. Thus, PA plus FP59 is extremely potent to all mammalian cells due to the ubiquitous expression of the PA receptors TEM8 (tumor endothelium marker-8) and CMG2 (Capillary morphogenesis protein-2) [30].

**Fig 1:**
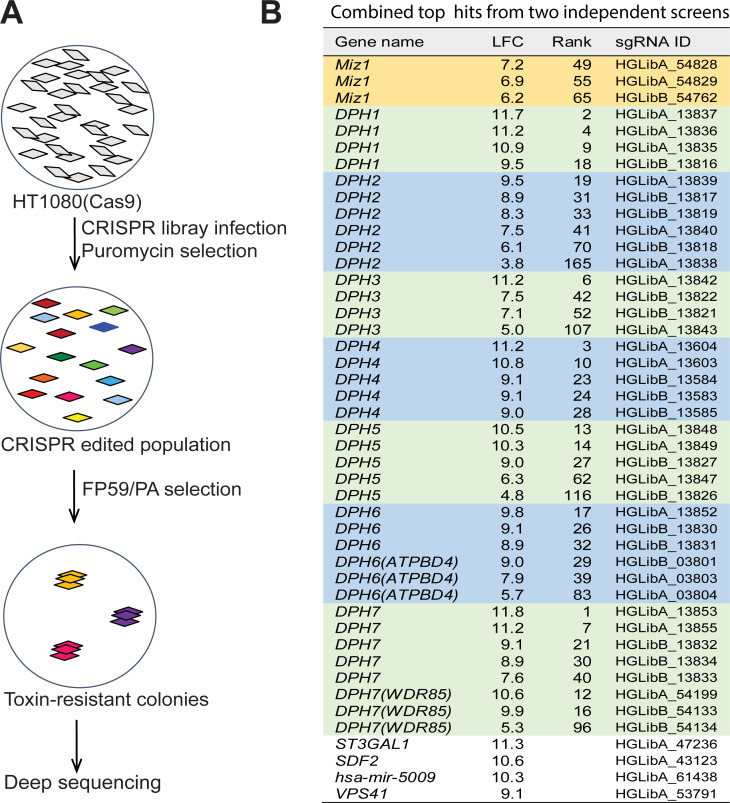
Whole genome CRISPR knockout screens to identify host factors required for the action of PA/FP59. **A**. A workflow of the whole genome CRISPR knockout screen. 6 × 10^7^ HT1080 (Cas9 expressing) cells were infected with the human lentiviral CRISPR library A or B (M.O.I. = 0.3). The CRISPR gene-edited cells were selected by puromycin (5 μg/ml) for 10 days. Then, the cells were treated with PA/FP59 (100 ng/ml each, about 10-fold of the minimum lethal concentration) twice with a two-day interval between treatments, and the toxin-resistant cells were allowed to form colonies by further culturing. The surviving, toxin-resistant cells were pooled, DNA extracted, and the sgRNA-containing fragments amplified and analyzed by Illumina deep sequencing for sgRNAs. **B**. Top hits from both library-A and library-B by Illumina deep sequencing. Only the genes with Log2(fold change) (LFC) ≥ 3 and were hit by ≥ 2 sgRNAs were considered as true positives and listed, along with the top four potential hits with just a single sgRNAs. Please refer to the S1 Data and S2 Data for details.

We performed two independent CRISPR KO screens in human HT1080 cells as outlined in Fig 1A, using the human CRISPR lentiviral pooled library-A and library-B [31, 32]. Each of these libraries covers 19,050 human genes, with each gene targeted by two different sets of three single-guide RNAs (sgRNAs), respectively [31, 32]. We hypothesized that the PA/FP59-resistant cells isolated after infections with the CRISPR lentiviral pooled libraries and the subsequent toxin exposure should be enriched for cells in which all the genes necessary for diphthamide biosynthesis had undergone sgRNA-mediated KO. Those genes should be identifiable by subsequent Illumina deep sequencing of the related sgRNAs in surviving cells. To achieve saturated targeting, in both screens we infected 60 million HT1080 cells at a multiplicity of infection of 0.3, ensuring the cells being targeted by each sgRNA 300 times (60 × 10^6^ × 0.3 divided by ~60,000 (the number of sgRNAs in each library)). We reasoned that a successful screen would identify majority of the factors required for diphthamide biosynthesis, which should include *Dph1* to *Dph7* and potential additional genes.

Interestingly, both library-A and library-B screens revealed that all seven *Dph* genes to be identified recurrently with ≥ 3 sgRNAs per gene (LFC (Log_2_[fold change]) ≥ 3; S1 Data, S2 Data, and Fig 1B), demonstrating the overall robustness of our CRISPR screens. Intriguingly, Miz1 (also named ZBTB-17), a BTB/POZ domain-containing transcription factor, was also observed in both screens with two sgRNAs in library-A screen and one sgRNA in our library-B screen (S1 Data, S2 Data, and Fig 1B). Notably, no other genes were observed with a frequency ≥ 2 sgRNAs even after combining the results from both screens (S1 Data, S2 Data, and Fig 1B). Other hits having only a single sgRNA are likely to be false positives, perhaps resulting from co-infection (being passengers) with one of the biologically active hits. To verify this and confirm the essential role of Miz1 in the toxin action, we regenerated the *Miz1*-, *Dph1*- (as a positive control), as well as the top four hits by single sgRNAs–*ST3GAL1*-, *SDF2*-, hsa-mir-5009-, and *VPS41*-gene edited HT1080 cells (Fig 1B) by CRISPR editing. We examined the toxin sensitivity of 20–30 clones of each of these CRISPR edited cells, and found that only Miz1- and Dph1-KO cells were resistant to PA/FP59 (S1 Table).

### Miz1 is required for diphthamide biosynthesis

**Fig 2:**
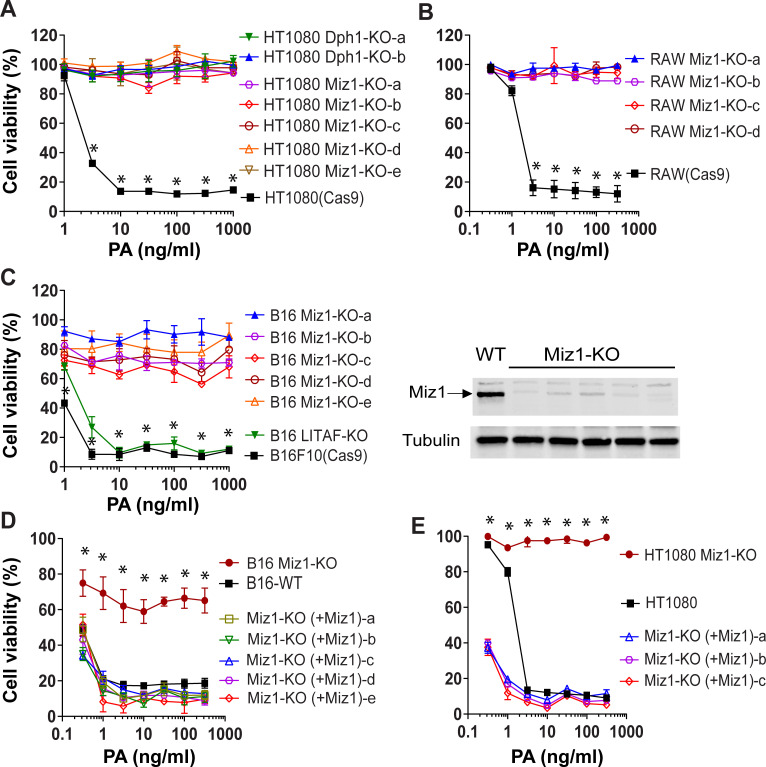
Miz1-decificent cells are resistant to PA/FP59. **A**-**C**. Knockout of Miz1 in HT1080 (A), RAW264.7 (B), and B16F10 (C) cells by CRISPR gene editing rendered these cells resistant to PA/FP59. The results from several independent clones are shown. In (A), knockout of Dph1 was used as an additional control. The indicated cells were incubated with various concentrations of PA in the presence of FP59 (100 ng/ml) for 48 h, followed by an MTT assay evaluating cell viability. Right panel in (C), lacking of Miz1 expression in representatives of B16 Miz1-KO clones was confirmed by Western blotting using an anti-Miz1 antibody (R&D Systems Cat. No. AF3760). Data are represented as mean (triplicate) ± SD. Unpaired two-tailed Student's *t*-tests, *, *p* < 0.001 between wild-type HT1080 (in A), RAW264.7 (in B), or B16F10 (in C) cells and their isogenic Miz1-KO or Dph1-KO cells. **D** and **E**. Reversion of the toxin-resistant phenotype of the B16 Miz1-KO cells (D) and HT1080 Miz1-KO cells (E) by transfection of a Miz1-expression plasmid. The results from several independent clones are shown. Cells were incubated with various concentrations of PA in the presence of FP59 (100 ng/ml) for 48 h, followed by an MTT assay evaluating cell viability. Of note, the Miz1-KO cells transfected with Miz1 regained their sensitivity to the toxin. Data are represented as mean (triplicate) ± SD. Unpaired two-tailed Student's *t*-tests, *, *p* < 0.001 between B16F10 Miz1-KO (in D) or HT1080 Miz1-KO (in E), and their isogenic Miz1 transfected cells.

To test whether Miz1 is generally required for the action of ADP-ribosylating toxins, we generated a Miz1-KO cell line from the mouse macrophage RAW264.7 cell line, as well as a Miz1-KO melanoma B16F10 cell line, using CRISPR gene editing. Similar to HT1080 cells, the two Miz1-KO cell lines generated above exhibited resistance to PA/FP59 in cytotoxicity assays (Fig 2A–2C). An example of confirmation of lacking Miz1 expression in Miz1-KO cells was shown by Western blotting analyses (Fig 2C, right panel). Importantly, the toxin-resistant phenotype of these cells could be completely reverted by complementation with Miz1 expression following transfection (Fig 2D and 2E). These results suggest a general requirement for Miz1 in the toxin’s action.

**Fig 3:**
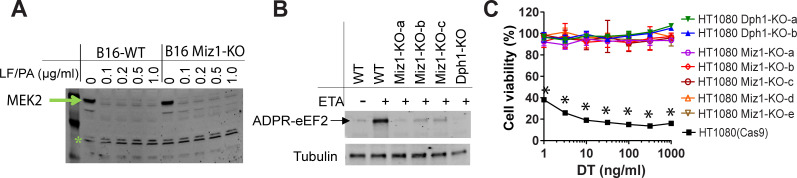
Miz1 is essential for diphthamide modification. **A**. Miz1 is not required for the toxin entry. B16F10 WT and Miz1-KO cells were incubated with various concentrations of PA/LF for 2 h, then cell lysates were analyzed by Western blotting using an antibody against the N-terminus of MEK2. Of note, LF could normally be delivered into B16 Miz1-KO cells, resulting in MEK2 cleavage (disappearance of MEK2). A non-specific band (indicated by *) served as a loading control. Representative of three independent experiments. **B**. eEF2 in Miz1-deficient B16F10 cells could not be ADP-ribosylated by ETA (FP59). The cell lysates from indicated cells were treated with FP59 in the presence of Biotin-NAD^+^ for 30 min and then analyzed by Western blotting using streptavidin-IR conjugate. Three independent B16F10 Miz1-KO clones were analyzed along with a B16F10 Dph1-KO clone as an additional control. Representative of three independent experiments. **C**. Miz1-deficient HT1080 cells were resistant to DT. HT1080 WT and Miz1-KO cells were incubated with various concentrations of DT for 48 h, followed by MTT assay evaluating cell viability. Five independent HT1080 Miz1-KO clones were analyzed along with two HT1080 Dph1-KO clones as additional controls. Data are represented as mean (triplicate) ± SD. Unpaired two-tailed Student's *t*-tests, *, *p* < 0.001 between wild-type HT1080 cells and their isogenic Miz1-KO or Dph1-KO cells.

To investigate whether Miz1 works in the early steps of toxin entry, we incubated B16F10 Miz1-KO cells with PA/LF and then examined for MEK2 cleavage, a known substrate of LF proteolytic activity [33]. We found that MEK2 from both WT cells and Miz1-KO cells could be equally and efficiently cleaved by LF (Fig 3A), demonstrating that cells lacking Miz1 can undergo normal toxin internalization.

Next, we explored whether Miz1-KO cells have a defect in diphthamide modification on eEF2 thereby making it unable to be ADP-ribosylated by ADPR toxins (S1 Fig). We prepared eEF2-containing cell lysates from B16F10 WT and Miz1-KO cells, and performed an ADP-ribosylation assay by incubation of these cell lysates with FP59 (ETA fusion) in the presence of biotin-labeled NAD^+^. Notably, ADP-ribosylation of eEF2 in Miz1-KO cells was diminished (Fig 3B), indicating that Miz1 is required for diphthamide modification on eEF2. Consistent with these observations, we also noted that human HT1080 Miz1-KO cells, which express the active DT receptor (human heparin-binding epidermal growth factor) (mouse cells do not express DT receptor), were also resistant to DT, another ADP-ribosylating toxin that targets diphthamide (Fig 3C).

### Miz1 is required for diphthamide modification via regulating Dph1 expression

**Fig 4:**
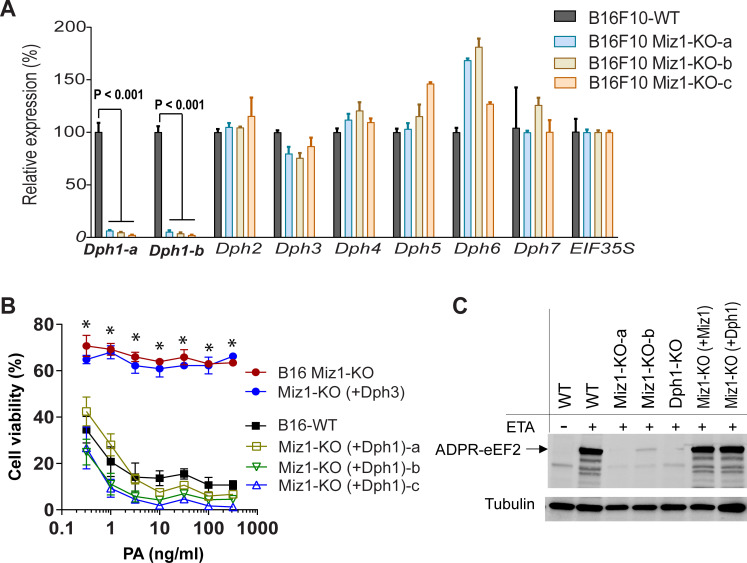
Miz1 is required for diphthamide biosynthesis through direct transcriptional regulation of Dph1 expression. **A**. Real-time PCR analyses of the seven known *Dph* genes’ expression in B16F10 WT and Miz1-KO cells. Eukaryotic translation initiation factor EIF35S was used as an internal normalization control. Of note, *Dph1* expression is diminished in all Miz1-KO cells. Two set of *Dph1* primers were used for a confirmational purpose (a, b). Three independent B16F10 Miz1-KO clones were analyzed. Data are represented as mean (triplicate) ± SD. Of note, B16F10 Miz1-KO cells exhibited consistent diminished *Dph1* expression. Unpaired two-tailed Student's *t*-tests. **B**. Exogenous expression of Dph1 in B16F10 Miz1-KO cells reverts their toxin-resistant phenotype. B16F10 Miz1-KO cells were transfected with Dph1- or Dph3-expressing plasmid. Cells were incubated with various concentrations of PA in the presence of FP59 (100 ng/ml) for 48 h, followed by an MTT assay evaluating cell viability. Three independent B16 Miz1-KO- (+Dph1) clones were analyzed. Data are represented as mean (triplicate) ± SD. Unpaired two-tailed Student's *t*-tests, *, *p* < 0.001 between B16F10 Miz1-KO cells and their isogenic *Dph1* transfected cells. **C**. eEF2 from B16F10 Miz1-KO cells regains the ADP-ribose acceptor ability via transfection with either a Dph1- or Miz1-expression construct. The indicated cell lysates were treated with FP59 (100 ng/ml) in the presence of biotin-labelled NAD^+^ for 30 min and then analyzed by Western blotting using streptavidin-IR conjugate. Representative of three independent experiments.

Miz1 is a transcription factor, thus is likely to be required for diphthamide biosynthesis by regulating one or more *Dph* gene expression. To test this, we surveyed the expression of the seven *Dph* gene in B16F10 cells and three independent B16F10 Miz1-KO clones by quantitative reverse transcriptase-PCR analyses. Markedly, *Dph1* expression was diminished in all Miz1-KO cells while other *Dph* genes were not markedly altered (Fig 4A). The role of Miz1 in *Dph1* expression was also confirmed by an analysis of human HT1080 WT cells and their corresponding isogenic Miz1-KO counterparts (S2 Fig). Based on these results, we reasoned that reconstitution of *Dph1* expression in Miz1-KO cells may bypass the requirement of Miz1 for endogenous Dph1 expression, correcting the diphthamide synthesis defect, and thereby reverting the toxin-resistant phenotype of the Miz1-KO cells. To test this, we transfected a Dph1-expressing construct into B16F10 Miz1-KO cells. We found that the exogenous expression of *Dph1* but not *Dph3* could completely rescued the toxin-resistant phenotype, as well as the ADP-ribose acceptor activity of eEF2 (Fig 4B and 4C).

**Fig 5:**
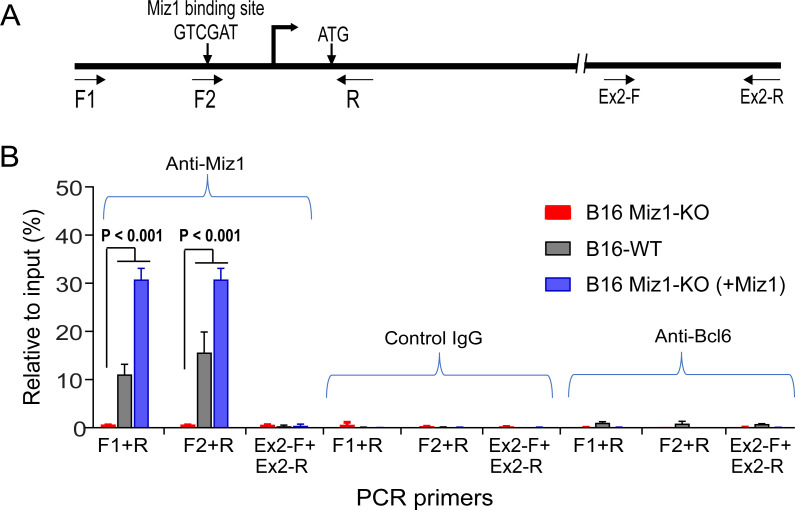
ChIP assay reveals the direct association of Miz1 and the *Dph1* promoter. **A**. The mouse *Dph1* proximal promoter contains a consensus Miz1-binding sequence. The locations of the transcription start site, initiation codon, and the primers used for the ChIP assay (in B) are shown. **B**. ChIP assay demonstrates the binding of Miz1 to the *Dph1* proximal promotor. Protein cross-linked chromatin DNA from the indicated cells was immunoprecipitated with an anti-Miz1 antibody, and was subjected to quantitative PCR analyses using the primers shown in (A). The same amounts of the chromatin DNA prior to immunoprecipitation was used as input controls. Of note, specific association of Miz1 and the *Dph1* promoter was detected only in B16F10 WT and Miz1-KO (+Miz1) cells, but not in Miz1-KO cells. The specificity of the anti-Miz1 antibody (R&D Systems Cat. No. AF3760) was validated by Western blotting analysis as shown in **Fig 2C**. Higher levels of amplification were detected in B16F10 Miz1-KO (+Miz1) cells were obviously due to the overexpression of the transfected Miz1. A control DNA fragment located in exon 2, which is approximately 0.8 Kb downstream of the Miz1-binding site was included as a PCR control. In additional to the isotype control antibody, an anti-Bcl6 (another BTB/POZ family member) antibody was included as an additional control. Data are represented as mean (triplicate) ± SD. Unpaired two-tailed Student's *t*-tests.

As a transcription factor, Miz1 was reported to directly bind to DNA via a consensus sequence to activate transcription [34]. To investigate whether Miz1 can directly bind to the proximal *Dph1* promoter, we first analyzed the *Dph1* promoter sequences from various species including human, mouse, rat, and Chinese hamster. Of note, we found that a consensus Miz1-binding site (A/GTCGAT) exists in all these proximal promoter sequences (S3A Fig). We then directly assessed the association of Miz1 and the *Dph1* promoter by performing a Chromatin Immunoprecipitation (ChIP) assay using B16F10 WT, Miz1-KO, and Miz1-KO (+Miz1) (transfected with a Miz1-expression plasmid) cells. We found that the *Dph1* promoter fragments surrounding the Miz1-binding site could be co-immunoprecipitated with Miz1 in B16F10 WT and Miz1-KO (+Miz1) cells but not in Miz1-KO cells (Fig 5).

Epigenetic methylation of the carbon-5 position of cytosine in CpG islands is often found in promoter regions as a common mechanism for suppression of gene expression. We assessed whether Miz1-binding is required for maintaining the active demethylation status of the proximal *Dph1* promoter using a bisulfite DNA sequencing approach [35]. Bisulfide treatment of genomic DNA can efficiently convert all cytosine residues to uracil but leaves 5-methylcytosine residues unaffected. We extracted the genomic DNA from both B16F10 WT cells and the corresponding Miz1-KO cells and performed bisulfite conversion, followed by PCR amplification and sequencing of the DNA fragments around the Miz1-binding site. We found that like WT B16F10 cells, the CpG islands in the Miz1-KO cells retained an un-methylated status (S3B Fig), suggesting that Miz1 ability to activates *Dph1* transcription is likely not through an alteration in the methylation of the *Dph1* promoter.

## Discussion

The essential diphthamide biosynthesis on eEF2 is one of the most complex post-translational modifications in eukaryotic cells. It has taken four decades to identify the seven non-redundant genes in diphthamide biosynthesis [5, 6, 18–23], but whether additional factors are required and how the pathway is regulated remained elusive. To address these issues, we performed two saturating, independent, and unbiased genome-wide CRISPR knockout screens. The screens concluded independently that Dph1 to Dph7 and additionally Miz1 are likely to be the majority of factors required for diphthamide biosynthesis; as no any other factors were identified (≥ 2 sgRNAs) even when we combined both screens. Therefore, while demonstrating the robustness of whole genome CRISPR-based screens for dissecting complex biological pathways, this work along with previous work also validates the value of this unbiased genetic approach in uncovering host factors required for pathogenesis of bacterial toxins [36].

There were a number of genes that play a role in anthrax toxin’s pathogenicity that were not uncovered in our screen. For instance, we used a modified anthrax lethal toxin for these screens, which needs the toxin receptor TEM8 or CMG2 to gain entry into target cells. The reason that we likely did not identify TEM8 or CMG2 from either screens is that HT1080 cells express both TEM8 and CMG2, with each receptor able to independently mediate toxin entry. Therefore, the genome-wide CRISPR KO screen is expected to only identify functionally non-redundant genes under the stringent selection conditions we used in this work (Fig 1). Since the toxin concentration (100 ng/ml each of PA and FP59) used in these screens was about 10-fold of its minimum lethal concentration (LC_100_) to the cells, and that inactivation of potential weaker regulators of the diphthamide pathway was unlikely to confer the extents of toxin resistance allowing cell survival through the screens, we expected to identify only the non-redundant essential factors and strong regulators of the pathway. As such, our screens appeared to have missed other potential modulators that regulate milder phenotypes or are essential for cell viability. We did not identify the othologue (SERGEF) of the yeast protein Kti13, which is involved in diphthamide modification in yeast via association with Kti11/Dph3 [37]. One possibility is that the Kti13 othologue may have other essential roles (such as tRNA modifications) in normal physiology. Acidification of endosomes by the vacuolar ATPase proton pump is also known to be required for ADPR toxins ETA, DT, and PA/FP59 to reach the cytosol of target cells [1, 29, 38, 39]. We did not however identify any genes for the various components of the endosomal acidification machinery. This is likely due to the essential role these genes play in overall cell viability. Supporting this notion, we found the cells CRISPR-edited for any of the ATPase proton pump components–*Atp6v1c1*, *Atp6v1e1*, *Atp6v1h*, *Ccdc115*, *Tmem199*, *Wdr7*, quickly lost viability during culture.

Miz1 is a BTB/POZ domain-containing transcription factor that is ubiquitously expressed. It was named Miz1 (Myc-interacting zinc finger protein 1), following the identification of its association with Myc, a basic helix-loop-helix transcription factor, in a two-hybrid analysis [40]. Myc is a well-characterized oncogene; and deregulation of c-Myc occurs in 50% of all human cancers [41, 42]. In the absence of Myc, Miz1 binds to the core promoter of Miz1 target genes and activates their expression in response to signals such as TGF-β [43–45], DNA damage [45], and inhibition of protein translation [46]. Among Miz1’s previously best characterized targets are those encoding the cyclin-dependent kinase inhibitors *p15Ink4b*, *p21Cip1*, and *p57Kip2* and a group of genes encoding proteins involved in cell-cell and cell-matrix adhesion [44, 45, 47], as well as genes required for autophagic flux [48]. However, in the presence of Myc, Miz1 shifts its activity from activation to repression of transcription [49].

In this work, we found that Miz1 is absolutely required for *Dph1* transcription via binding to a consensus sequence in *Dph1* core promoter region in mammals. This not only reveals a novel function of Miz1, but also provides critical insights into understanding the regulation of diphthamide biosynthesis (S1 Fig). Thus, Miz1 would also be likely essential to the established roles of diphthamide in ensuring the fidelity of mRNA translation, maintaining cellular fitness and adaptation to various stresses [12, 13–17]. Similar as other *Dph* gene KO cells [5, 6, 12], the Miz1-KO cells generated in this study do not exhibit obvious proliferative defects. However, Miz1 is essential in normal development; as Miz1-KO mice are lethal at early stages of development [50, 51]. This might be due, at least partially, to a defect in diphthamide modification on eEF2. As mentioned previously, other *Dph* gene knockout mice are also embryonic lethal [9–12]. Mutations in Miz1 are found to cause human cardiomyopathy and heart failure. Whether the diphthamide defects caused by these Miz1 mutations contribute to the etiology of these diseases remains to be investigated. While diphthamide biosynthesis machinery (Dph1-Dph7) exists across eukaryotes, NCBI BLAST search could not identify Miz1 orthologues in the lower species such as yeast, C. elegans, and Drosophila. Interestingly, Miz1 exists in the small vertebrate zebrafish, indicating that Miz1 emerged much later in evolution. It would be interesting to investigate in future whether Miz1 is also required for Dph1 expression in zebrafish cells. Since the lack of Miz1 in cultured cells leads to nearly complete loss of Dph1 expression, we reason that the Miz1 absence may favor the occupancy of the Dph1 core promoter by an unidentified strong transcriptional repressor which may have coevolved with Miz1.

In summary, *Dph1*-7 and *Miz1* are likely to be the major non-redundant factors required for the biosynthesis/regulation of diphthamide on eEF2. Identification of the essential role of Miz1 in diphthamide biosynthesis opens a new avenue for understanding the role that diphthamide modification plays in normal physiology and bacterial toxin pathogenesis.

## Methods

### Cells and cytotoxicity assay

HT1080, RAW264.7, and B16F10 cells were grown in DMEM supplemented with 10% fetal bovine serum (FBS). For cytotoxicity assay, cells grown in 96-well plates (30~50% confluence) were incubated with various concentrations of PA (0–1000 ng/ml) in the presence of 100 ng/ml FP59 for 48 h. Cell viabilities were then assayed by an MTT (3-[4,5-dimethylthiazol-2-yl]-2,5-diphenyltetrazolium bromide, Sigma Cat. No. M5655) assay as described previously [29], expressed as % of signals of untreated cells. For toxin entry assay, cells were incubated with various concentrations of PA/LF for 2 h. Then cell lysates were prepared in the modified RIPA lysis buffer containing protease inhibitors [29]. Cell lysates were separated on SDS-PAGE gels, transferred onto nitrocellulose membranes, and analyzed by Western blotting using an anti-MEK2 antibody (Santa Cruz Biotechnology, sc-524).

PA, LF, and FP59 were purified as described previously (Liu et al., 2007; Gupta et al., 2008; Firoved et al., 2005) [52].

### CRISPR pooled lentiviral library screen and CRISPR gene editing

The genome-wide CRISPR knockout lentiviral pooled human library-A and library-B developed by Feng Zhang’s MIT laboratory [31] were used for our unbiased screens for host genes required for the toxin activity (Addgene Cat. # 1000000049). Each of these libraries contains three independent sgRNAs for each of the 19,050 human genes. Because the libraries are in the format of a two vector system, meaning Sp Cas9 is encoded by a separate vector, we first transfected lentiCas9-Blast plasmid (Addgene #52962) into HT1080 cells used for the screen. We followed the protocols provided by Addgene and in reference [31] for the pooled lentiviral library preparations and infections. Briefly, the library DNA was packaged to form pooled lentiviral sgRNA libraries and titrated. For each library screen, 60 million HT1080 (Cas9-expressing) cells were infected with the lentiviral pooled library (A or B) at a M.O.I. (virus/cell ratio) of 0.3, covering the library ~300 times. Infected cells were divided into twelve 15-cm (diameter) culture dishes and selected with puromycin (5 μg/ml) for three days. Cells were passed by trypsinization to maintain 50% confluence for one week allowing the completion of the gene editing process. Then for each cell plate, half of the cells were frozen for the later genomic DNA isolation as the non-selected control, and half of the cells were cultured and subjected to PA/FP59 selection. In brief, cells were incubated with PA/FP59 (100 ng/ml each, ~10 times of the minimum concentration needed to kill 100% of the cells) for 48 h. Dead cells were removed by replacing with fresh medium. Two days later, the survivors were treated again with PA/FP59 (100 ng/ml each) for 48 h. Surviving cells were collected into four different pools for genomic DNA isolation and sgRNA DNA amplification by two rounds of PCR using the primers listed in S2 Table. The PCR products were sequenced on Illumina Hiseq3000 for single end 55 bps using standard Illumina sequencing primer with 10% PhiX to further balance sequence diversity. The raw sequencing data trimmed off the adaptor sequence were matched to the guide sequences from the filtered library files using the Model-based Analysis of Genome-wide CRISPR/Cas9 Knockout (MAGeCK) algorithm (v0.5.6) count function [53]. Normalized sgRNA counts from the toxin-resistant cells over non-toxin selected cells were calculated, and expressed as LFC (Log2[fold change]). Read counts were subjected to MAGeCK *mle* analysis module, to obtain p values, FDRs (or adjusted p values) [53]. Genes over-represented at an FDR < 0.1 and LFC ≥ 3 were considered as hits.

To generate HT1080, RAW264.7, and B16F10 CRISPR edited cells, we cloned the indicated sgRNA sequences (S2 Table) into the pSpCas9-2A-puro vector (Addgene, PX458). We transfected the resulting sgRNA constructs into the studied cells, using X-tremeGENE 9 DNA Transfection Reagent following the manufacturer’s manual (Roche, Cat. No. 06366236001). The transfected cells were cultured for 10 days in the presence of 5 μg/ml puromycin, allowing formation of individual colonies. The gene-editing was confirmed by DNA sequencing or Western blotting analyses.

### Reverse transcriptase PCR and gene expression constructs

Total RNA was prepared from the indicated cells using TRIzol reagent (Invitrogen, Carlsbad, CA), and was used to synthesize single-strand cDNA using the SuperScript IV First-Strand Synthesis System following the manufacturer’s manual (Invitrogen, Cat. No. 18091050). Full-length Miz1 cDNA was amplified by reverse transcriptase-PCR from HT1080 cells, cloned into pIREShyg-2 mammalian expression vector, and confirmed by DNA sequencing. The primers used for cloning are listed in S2 Table. The Dph1 and Dph3 expressing plasmids used in this work were described previously [5, 6]. X-tremeGENE 9 DNA Transfection Reagent was used for transfection of the plasmids into the indicated cells following the manufacturer’s manual (Roche, Cat. No. 06366236001).

### ChIP assay

B16F10 WT, Miz1-KO, and Miz1-KO (+Miz1) cells were cross-linked with 1% formaldehyde at 37°C for 10 min. Sonication was carried out after swelling in a Bransson sonifier until the majority of fragments show nucleosomal size. An anti-Miz1 antibody (R&D Systems Cat. No. AF3760) coupled to Protein G-Dynabeads (Invitrogen) was used for immunoprecipitation. DNA was purified with Qiagen PCR purification kit after elution of the bound chromatin with 1% SDS and reversion of the crosslink. The control antibodies included an isotype control IgG (goat anti-HXK II, Santa Cruz Biotechnology Cat. No. sc-6521) and an anti-Bcl6 (Cell Signaling Technology Cat. No. 14895). The DNA was used for quantitative PCR analyses of the *Dph1* promoter sequences around the Miz1 consensus binding site or a fragment in the exon 2 using the primers listed in S2 Table.

### Bisulfite sequencing

Bisulfite sequencing analysis was performed using the EpiJET Bisulfite Conversion Kit (Thermo Scientific Cat. No. K1461). Two μg of genomic DNA was subjected to bisulfite conversion following the manufacturer’s manual. The primers avoiding CpG islands-containing regions as listed in S2 Table were used for PCR amplification and sequencing of the bisulfite converted fragments surrounding the Miz1 consensus site in the mouse *Dph1* proximal promoter.

### ADP-ribosylation assay

Cell extracts were prepared in the modified RIPA buffer containing protease inhibitors as described previously (Liu and Leppla, 2003). ADP-ribosylation reactions were performed at 37°C for 1 hour in a volume of 50 μl ADP-ribosylation buffer (20 mM Tris-HCl, pH 7.4; 1 mM EDTA; 50 mM DTT) containing 50 μg of extract, 100 ng FP59, and 10 μM 6-Biotin-17-NAD^+^ (Trevigen). Samples were then mixed with a SDS sample buffer, boiled for 5 min and separated on 4−25% SDS-PAGE gels (Invitrogen). The proteins were transferred to nitrocellulose membranes and Western blotting was performed using streptavidin-IR conjugate (Rockland Immunochemicals, Gilbertsville, PA).

### Statistical analysis

Cell culture experiments were repeated a minimum of three times in triplicate. In comparisons between two groups with equal variance, unpaired two-tailed Student's *t*-tests was used to identify significant (*P* < 0.05) differences.

## Supporting information

S1 Fig Factors required for diphthamide biosynthesis pathway. Diphthamide biosynthesis is carried out in four steps by the seven highly conserved proteins Dph1-7 from yeast to humans. Step1: transfer of the 3-amino-3-carboxypropyl (ACP) group from S-adenosyl methionine (AdoMet) to the imidazole ring of His^715^ residue (in all mammals) of eEF2 by Dph1 to Dph4, yielding ACP-eEF2. Step 2: the ACP-eEF2 undergoes tetra-methylation by Dph5, yielding the methylated diphthine. Step 3: Dph7 catalyzes the demethylation, generates diphthine. Step 4: diphthine is converted to diphthamide by amidase Dph6. N-1 (arrow head) of the histidine imidazole ring of diphthamide is the site for ADP-ribosylation by DT, ETA and other ADRTs. Ado-S-Me, methylthioadenosine; Ado-Hcy, S-adenosylhomocysteine; NAD^+^, nicotinamide adenine dinucleotide. In “ADP-ribosyl diphthamide”: A, adenine moiety; R, ribosyl moiety.
(EPS)

S2 Fig Real-time PCR analyses of the seven known *Dph* genes’ expression in HT1080 WT and Miz1-KO cells. Eukaryotic translation initiation factor EIF35S was used as an internal normalization control. Of note, *Dph1* expression is diminished in all Miz1-KO cells. Three independent HT1080 Miz1-KO clones were analyzed. Note, HT1080 Miz1-KO cells exhibited consistent diminished *Dph1* expression. Data are represented as mean (triplicate) ± SD. Unpaired two-tailed Student's *t*-tests.
(EPS)

S3 Fig The *Dph1* proximal promoters and bisulfite-sequencing analyses. **A**. DNA sequence alignment of the *Dph1* proximal promoters from human, mouse, rat, and Chinese hamster. The alignment was generated using the EMBL-EBL MUSCLE program (https://www.ebi.ac.uk/Tools/msa/muscle/). A consensus Miz1 binding site (A/GTCGAT, in blue) exists in these highly homologous *Dph1* promoter sequences. Start codon were labeled in green. * indicates the same residue. **B**. The CpG islands in mouse *Dph1* proximal promoter are not methylated in both B16F10 WT cells and Miz1-KO cells. Bisulfite-sequencing examples of a promoter fragment from the B16F10 WT cells and Miz1-KO cells are shown. The original unconverted sequence and theoretically converted sequence except for the CpG islands are also shown at the bottom. Of note, all the cytosine residues in the indicated CpG islands were converted to thymidine residues.
(EPS)

S1 Data Illumina deep sequencing counts of sgRNAs in HT1080(Cas9) cells infected with the CRISPR lentiviral pooled library-A after PA/FP59 selection (only sgRNAs with LFC ≥ 3 listed). (XLSX)

S2 Data Illumina deep sequencing counts of sgRNAs in HT1080(Cas9) cells infected with the CRISPR lentiviral pooled library-B after PA/FP59 selection (only sgRNAs with LFC ≥ 3 listed). (XLSX)

S3 Data Supporting numerical data underlying graphs. (XLSX)

S1 Table Toxin sensitivity of HT1080 cells edited by the indicated sgRNAs. (DOCX)

S2 Table Primers used for cloning, CRISPR editing, and qPCR. (DOCX)
