# Supplementary material for: Identification of the transcription factor Miz1 as an essential regulator of diphthamide biosynthesis using a CRISPR-mediated genome-wide screen
Source: PLoS Genet. 2020 Oct 15;16(10):e1009068. doi: 10.1371/journal.pgen.1009068 (PMC7591051; doi:10.1371/journal.pgen.1009068)
Supplement: S1 Table — (DOCX) [file pgen.1009068.s007.docx]

**S1 Table**. Toxin sensitivity of HB1080 cells edited by the indicated sgRNAs

| sgRNA | Toxin^R^ vs. Total clones (%) |
| --- | --- |
| ZBTB17-a | 19/21 (90%) |
| ZBTB17-b | 19/21 (90%) |
| Dph1 | 23/24 (96%) |
| ST3GAL1 | 0/30 (0%) |
| SDF2 | 0/27 (0%) |
| hsa-mir-5009 | 0/20 (0%) |
| VPS41 | 0/30 (0%) |

The independent CRISPR-edited HT1080 clones were incubated with PA/FP59 (100 ng/ml each) for 48 h. The clones with > 50% viable cells were considered as toxin resistant clones (Toxin^R^).
