# Supplementary material for: Identification of the transcription factor Miz1 as an essential regulator of diphthamide biosynthesis using a CRISPR-mediated genome-wide screen
Source: PLoS Genet. 2020 Oct 15;16(10):e1009068. doi: 10.1371/journal.pgen.1009068 (PMC7591051; doi:10.1371/journal.pgen.1009068)
Supplement: S2 Table — (DOCX) [file pgen.1009068.s008.docx]

**S2 Table**. Primers used for cloning, CRISPR editing, and qPCR

| Use | Primer name and sequence | | Note |
| --- | --- | --- | --- |
| Human Miz1 cDNA cloning | For | AAACTTAAGATGGACTTTCCCCAGCACAGCCAGCATGTCTT | 2421 bp, cloned into AflII/EcoRV sites of pIRESHgy2 |
|  | Rev | AACGTACGATATCTCACTCGGCAGGCGGGGGACATTCAGGAGCT |  |
| Human Miz1  CRISPR editing | For | CACCGCGAGGCGCGCTACCGCTGCG | After annealing, cloned into BpiI-digested pSpCas9-based vectors |
|  | Rev | AAACCGCAGCGGTAGCGCGCCTCGC |  |
| Human Miz1  CRISPR editing | For | CACCGGCGCTTGAGGTTGCCCGAGG | Same as above |
|  | Rev | AAACCCTCGGGCAACCTCAAGCGCC |  |
| Human Dph1  CRISPR editing | For | CACCGTGATGGGTGACGTGACCTAC | Same as above |
|  | Rev | AAACGTAGGTCACGTCACCCATCAC |  |
| Mouse Miz1  CRISPR editing | For | CACCGGGACTCAGTGCGATCCCCGT | Same as above |
|  | Rev | AAACACGGGGATCGCACTGAGTCCC |  |
| Mouse Miz1  CRISPR editing | For | CACCGGGAGGGTCAGAACCTGCGCT | Same as above |
|  | Rev | AAACAGCGCAGGTTCTGACCCTCCC |  |
| Mouse Dph1  CRISPR editing | For | CACCGGTCTTGAACGGAGGTGTCCA | Same as above |
|  | Rev | AAACTGGACACCTCCGTTCAAGACC | Same as above |
| 1^st^-PCR for Illumina deep sequencing | For-1st | AATGGACTATCATATGCTTACCGTAACTTGAAAGTATTTCG | Products as templates for 2^nd^ PCR |
|  | Rev-1st | CTTTAGTTTGTATGTCTGTTGCTATTATGTCTACTATTCTTTCC |  |
| 2^nd^ PCR  for Illumina deep sequencing | For-2nd | 5’AATGATACGGCGACCACCGAGATCTACACTCTTTCCCTACACGACGCTCTTCCGATCT(1-9 nt stagger)tcttgtggaaaggacgaaacaccg3’ | Primers with 4 barcodes (in red) for 4 pooled of samples, respectively. |
|  | R-1 | 5’CAAGCAGAAGACGGCATACGAGATAAGTAGAGGTGACTGGAGTTCAGACGTGTGCTCTTCCGATCTTCTACTATTCTTTCCCCTGCACTGT3’ |  |
|  | R-2 | 5’CAAGCAGAAGACGGCATACGAGATACACGATCGTGACTGGAGTTCAGACGTGTGCTCTTCCGATCTTCTACTATTCTTTCCCCTGCACTGT3’ |  |
|  | R-3 | 5’CAAGCAGAAGACGGCATACGAGATCGCGCGGTGTGACTGGAGTTCAGACGTGTGCTCTTCCGATCTTCTACTATTCTTTCCCCTGCACTGT3’ |  |
|  | R-4 | 5’CAAGCAGAAGACGGCATACGAGATCATGATCGGTGACTGGAGTTCAGACGTGTGCTCTTCCGATCTTCTACTATTCTTTCCCCTGCACTGT3’ |  |
| Mouse Dph1 qPCR | For-1 | CAAGAAGGTGGCCTTACAAATGCCAGA | 187 bp |
|  | Rev-1 | CAGGAGCTGAAAGCTGATTATCACATC |  |
| Mouse Dph1 qPCR | For-2 | GCTGTGACCATAGTGCACCAGGAAGTCA | 163 bp |
|  | Rev-2 | GGTTGGCAATCATGACAGACTCCAGA |  |
| Mouse Dph2 qPCR | For | ACACAGCTTATGGCAGCTGCTGTGTGGAT | 166 bp |
|  | Rev | GCCTTTGCACAGAGCTCTAAAGCAACG |  |
| Mouse Dph3 qPCR | For | TTCACGACGAGGTGGAGATCGAGGACT | 229 bp |
|  | Rev | ACTAACTCCTTGTTGGTTGAAGGTGCT |  |
| Mouse Dph4 qPCR | For | TGCAGACCCATCTGCAAATATGTCAGA | 163 bp |
|  | Rev | ATTCCCTAGAATTTTCCATGCCTGATCA |  |
| Mouse Dph5 qPCR | For | GGGAGATGCCAAGGACATCACAGTCAA | 213 bp |
|  | Rev | AGGAACGCAACATCACTGACATCTGCA |  |
| Mouse Dph6 qPCR | For | ACAGCTGTTACAACATGATGCAGTGCA | 221 bp |
|  | Rev | ATAAACTCTTCCTGTCTCCAAGCTCCT |  |
| Mouse Dph7 qPCR | For | TGTGTGGAGGCTCAAGTGGCACCCAGT | 158 bp |
|  | Rev | CATATACTAATGAGTTAGGCATTTCGT |  |
| Human Dph1 qPCR | For | AAGAAGGTGGCCTTGCAAATG | 178 bp |
|  | Rev | GTAGTGCACCAAGAAGTCAGCT |  |
| Human Dph2 qPCR | For | GCAGCCTCATTCCTTAGTTCC | 125 bp |
|  | Rev | CCGCTTCCCTCATCCTCATAG |  |
| Human Dph3 qPCR | For | ATGGCAGTGTTTCATGACGAGGT | 249 bp |
|  | Rev | TCAGCATTTAACTAATTCTTTGTTGGC |  |
| Human Dph4 qPCR | For | GCTGCAGTCGAGTGTATCTGGAAG | 163 bp |
|  | Rev | AATGCAACATCACTGATATCAGCAT |  |
| Human Dph5 qPCR | For | CCAAGCAGCCCAGCAGCTTCT | 163 bp |
|  | Rev | CAAGTCCACAGTGCACATTTGC |  |
| Human Dph6 qPCR | For | CTAAGAAGTATGGAGTACATGT | 144 bp |
|  | Rev | AAGCCACAGGTGCAAATGCATCAG |  |
| Human Dph7 qPCR | For | TGTCATGAATGCAGAGAGGATAACG | 146 bp |
|  | Rev | TCTGGGTTCACGCCACAGTCACGT |  |
| ChIP Dph1 promoter | For-1 | TTGTACCAACCGGTGCCCATTGCAGCA | F1+R=193 bp  F2+R=110 bp |
|  | For-2 | GGCTCGGCGGTCGATTGTGTTACC |  |
|  | Rev | CAGTCTCGGACACAACCAGCGCCGCCAT |  |
| ChIP Dph1 exon 2 | For | GCAGGTGCTGGAGTTCATGTACACTGCT | 183 bp |
|  | Rev | CCTTCCACAGCTGAGGCGTCTGCGCTCT |  |
| Bisulfite PCR and sequencing | For1 | GTAGAATATTTTTAGGTATATTTATT | 441 bp |
|  | Rev1 | CCCTATCCCTAACCTAAAAATCCCACCAA |  |
|  | For2 | TTTTGAATTAGGTTAGAGAAGAG | 318 bp |
